# Crystal structure of 2,5-di­methyl­anilinium salicylate

**DOI:** 10.1107/S2056989015014401

**Published:** 2015-08-06

**Authors:** A. Mani, P. Praveen Kumar, G. Chakkaravarthi

**Affiliations:** a Department of Physics, Sri Venkateswaraa College of Technology, Sriperumbudur 602 105, India; b Department of physics, Presidency College, Chennai 600 005, India; c Department of Physics, CPCL Polytechnic College, Chennai 600 068, India

**Keywords:** crystal structure, hydrogen bonding, aromatic π–π stacking inter­actions

## Abstract

The title mol­ecular salt, C_8_H_12_N^+^·C_7_H_5_O_3_^−^ arose from the proton-transfer reaction between 2,5-xylidine and salicylic acid. In the anion, the dihedral angle between the planes of the aromatic ring and the –CO_2_^−^ group is 11.08 (8)°; this near planarity is consolidated by an intra­molecular O—H⋯O hydrogen bond. In the crystal, the components are connected by N—H⋯O hydrogen bonds, with all three O atoms in the anion acting as acceptors; the result is a [100] chain. The structure also features weak C—H⋯O bonds and aromatic π–π stacking [centroid-to-centroid distance = 3.7416 (10) Å] inter­actions, which lead to a three-dimensional network.

## Related literature   

For related structures, see: Fun *et al.* (2011 ▸); Mathlouthi *et al.* (2014 ▸); Smirani & Rzaigui (2009 ▸).
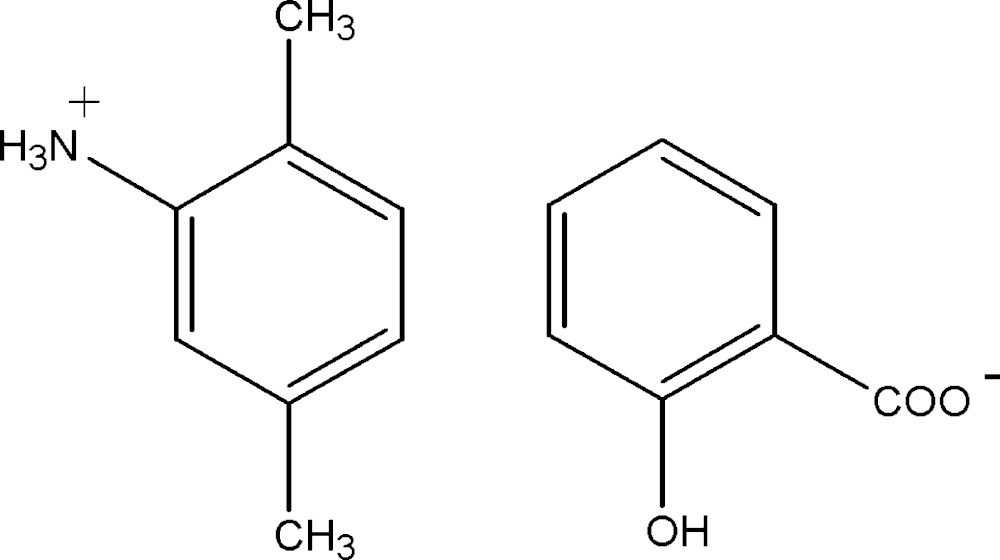


## Experimental   

### Crystal data  

C_8_H_12_N^+^·C_7_H_5_O_3_^−^
*M* *_r_* = 259.30
Monoclinic, 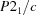

*a* = 6.9645 (5) Å
*b* = 20.6924 (14) Å
*c* = 9.2920 (7) Å
β = 95.738 (3)°
*V* = 1332.38 (17) Å^3^
*Z* = 4
Mo *K*α radiation
μ = 0.09 mm^−1^
*T* = 295 K
0.26 × 0.24 × 0.20 mm

### Data collection  

Bruker Kappa APEXII CCD diffractometer
Absorption correction: multi-scan (*SADABS*; Sheldrick, 1996 ▸) *T*_min_ = 0.977, *T*_max_ = 0.982
17007 measured reflections
3384 independent reflections
2339 reflections with *I* > 2σ(*I*)
*R*_int_ = 0.030

### Refinement  

*R*[*F* ^2^ > 2σ(*F* ^2^)] = 0.047
*wR*(*F* ^2^) = 0.134
*S* = 1.01
3384 reflections
178 parameters
1 restraint
H atoms treated by a mixture of independent and constrained refinement
Δρ_max_ = 0.22 e Å^−3^
Δρ_min_ = −0.21 e Å^−3^

### 

Data collection: *APEX2* (Bruker, 2004 ▸); cell refinement: *SAINT* (Bruker, 2004 ▸); data reduction: *SAINT*; program(s) used to solve structure: *SHELXS97* (Sheldrick, 2008 ▸); program(s) used to refine structure: *SHELXL97* (Sheldrick, 2008 ▸); molecular graphics: *PLATON* (Spek, 2009 ▸); software used to prepare material for publication: *SHELXL97*.

## Supplementary Material

Crystal structure: contains datablock(s) global, I. DOI: 10.1107/S2056989015014401/hb7474sup1.cif

Structure factors: contains datablock(s) I. DOI: 10.1107/S2056989015014401/hb7474Isup2.hkl

Supporting information file. DOI: 10.1107/S2056989015014401/hb7474Isup3.cml

**Fig. 1.**
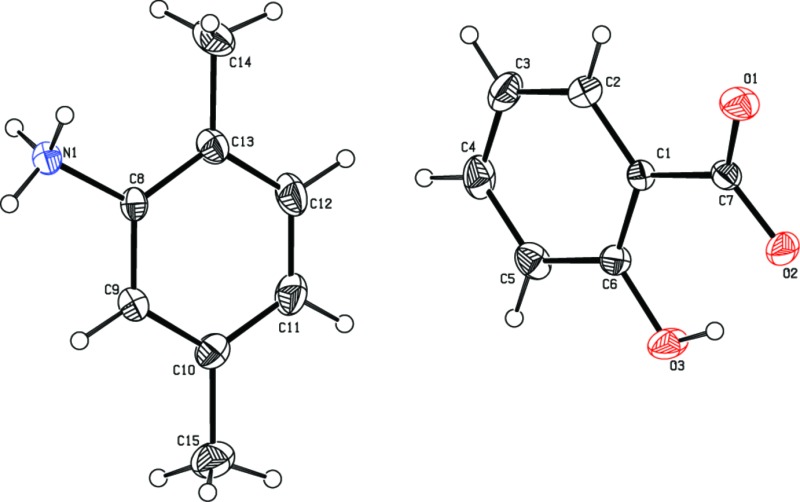
The molecular structure of (I), with 30% probability displacement ellipsoids for non-H atoms.

**Fig. 2.**
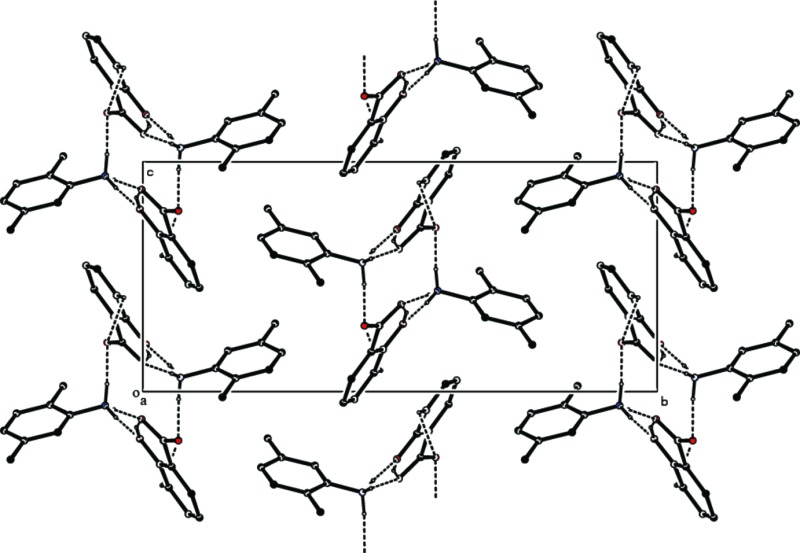
The packing of (I), viewed down a axis. Hydrogen bonds are shown as dashed lines. H atoms not involved in hydrogen bonding have been omitted for clarity.

. DOI: 10.1107/S2056989015014401/hb7474fig1.tif
The mol­ecular structure of (I), with 30% probability displacement ellipsoids for non-H atoms.

a . DOI: 10.1107/S2056989015014401/hb7474fig2.tif
The packing of (I), viewed down *a* axis. Hydrogen bonds are shown as dashed lines. H atoms not involved in hydrogen bonding have been omitted for clarity.

CCDC reference: 1415922

Additional supporting information:  crystallographic information; 3D view; checkCIF report

## Figures and Tables

**Table 1:** Hydrogen-bond geometry (, )

*D*H*A*	*D*H	H*A*	*D* *A*	*D*H*A*
O3H3*A*O2	0.83(1)	1.77(1)	2.5282(15)	151(2)
N1H1*A*O1^i^	0.89	1.80	2.6809(17)	169
N1H1*B*O2^ii^	0.89	1.92	2.7998(16)	168
N1H1*C*O3^iii^	0.89	2.08	2.9654(17)	171
C5H5O1^iv^	0.93	2.58	3.237(2)	128

Symmetry codes: (i) 
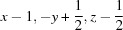
; (ii) 
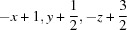
; (iii) 
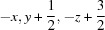
; (iv) 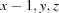.

## supplementary crystallographic information

### S1. Structural commentary

We herewith report the crystal structure of the title compound, (I), (Fig. 1). The geometric parameters of the title compound (I) (Fig. 1) are comparable with the reported structures [Fun *et al.*, 2011; Mathlouthi *et al.*, 2014; Smirani & Rzaigui,(2009)].

The conformation of the anion is stabilized by a weak O—H···O (Table 1) hydrogen bond. In the crystal structure, the adjacent anions and cations are linked by medium-strength N—H···O (Table 1) hydrogen bonds which link the anions and cations into infinite chain along [100] and these chains are further influenced by C—H···O hydrogen bond (Table 1 & Fig. 2) and π–π [Cg2···Cg2^i^ distance 3.7416 (10)Å; (i) 1-x, -y,1-z; Cg2 is the centroid of (C1—C6) ring] inter­actions to form a three dimensional network.

### S2. Synthesis and crystallization

A mixture of 2,5-xylidine and salicylic acid dissolved in ethanol (molar ratio 1:1:1) was stirred for 3 h and then kept at room temperature. The saturated solution was allowed to evaporating slowly at room temperature. After the evaporation period of three weeks, colourless blocks were recovered.

### S3. Refinement

H atoms were positioned geometrically and refined using riding model with C—H = 0.93 Å and Uiso(H) = 1.2Ueq(C) for CH, N—H = 0.89Å and Uiso(H) = 1.5Ueq(N) for NH_3_, C—H = 0.96Å and Uiso(H) = 1.5Ueq(C) for CH_3_. H atom for hydroxyl group was fixed from Fourier map and refined with Uiso(H) = 1.5Ueq(O) and O—H distance was restraint to 0.82 (1)Å.

### Crystal data

**Table:**

C_8_H_12_N^+^·C_7_H_5_O_3_^−^	*F*(000) = 552
*M**_r_* = 259.30	*D*_x_ = 1.293 Mg m^−^^3^
Monoclinic, *P*2_1_/*c*	Mo *K*α radiation, λ = 0.71073 Å
Hall symbol: -P 2ybc	Cell parameters from 4550 reflections
*a* = 6.9645 (5) Å	θ = 2.4–28.1°
*b* = 20.6924 (14) Å	µ = 0.09 mm^−^^1^
*c* = 9.2920 (7) Å	*T* = 295 K
β = 95.738 (3)°	Block, colourless
*V* = 1332.38 (17) Å^3^	0.26 × 0.24 × 0.20 mm
*Z* = 4	

### Data collection

**Table:**

Bruker Kappa APEXII CCD diffractometer	3384 independent reflections
Radiation source: fine-focus sealed tube	2339 reflections with *I* > 2σ(*I*)
Graphite monochromator	*R*_int_ = 0.030
ω and φ scan	θ_max_ = 28.7°, θ_min_ = 2.0°
Absorption correction: multi-scan (*SADABS*; Sheldrick, 1996)	*h* = −8→9
*T*_min_ = 0.977, *T*_max_ = 0.982	*k* = −27→27
17007 measured reflections	*l* = −12→12

### Refinement

**Table:**

Refinement on *F*^2^	Primary atom site location: structure-invariant direct methods
Least-squares matrix: full	Secondary atom site location: difference Fourier map
*R*[*F*^2^ > 2σ(*F*^2^)] = 0.047	Hydrogen site location: inferred from neighbouring sites
*wR*(*F*^2^) = 0.134	H atoms treated by a mixture of independent and constrained refinement
*S* = 1.01	*w* = 1/[σ^2^(*F*_o_^2^) + (0.0641*P*)^2^ + 0.3396*P*] where *P* = (*F*_o_^2^ + 2*F*_c_^2^)/3
3384 reflections	(Δ/σ)_max_ < 0.001
178 parameters	Δρ_max_ = 0.22 e Å^−^^3^
1 restraint	Δρ_min_ = −0.21 e Å^−^^3^

### Special details

**Table:**

Geometry. All esds (except the esd in the dihedral angle between two l.s. planes) are estimated using the full covariance matrix. The cell esds are taken into account individually in the estimation of esds in distances, angles and torsion angles; correlations between esds in cell parameters are only used when they are defined by crystal symmetry. An approximate (isotropic) treatment of cell esds is used for estimating esds involving l.s. planes.
Refinement. Refinement of F^2^ against ALL reflections. The weighted R-factor wR and goodness of fit S are based on F^2^, conventional R-factors R are based on F, with F set to zero for negative F^2^. The threshold expression of F^2^ > 2sigma(F^2^) is used only for calculating R-factors(gt) etc. and is not relevant to the choice of reflections for refinement. R-factors based on F^2^ are statistically about twice as large as those based on F, and R- factors based on ALL data will be even larger.

### Fractional atomic coordinates and isotropic or equivalent isotropic displacement parameters (Å^2^)

**Table:**

	*x*	*y*	*z*	*U*_iso_*/*U*_eq_	
C1	0.52627 (19)	0.05604 (7)	0.67259 (15)	0.0271 (3)	
C2	0.5588 (2)	0.09450 (8)	0.55505 (17)	0.0371 (4)	
H2	0.6825	0.1100	0.5463	0.045*	
C3	0.4109 (3)	0.11016 (9)	0.45114 (18)	0.0474 (4)	
H3	0.4352	0.1350	0.3715	0.057*	
C4	0.2267 (3)	0.08870 (9)	0.46651 (18)	0.0467 (4)	
H4	0.1260	0.1002	0.3979	0.056*	
C5	0.1897 (2)	0.05073 (9)	0.58152 (18)	0.0405 (4)	
H5	0.0646	0.0369	0.5911	0.049*	
C6	0.3394 (2)	0.03300 (7)	0.68349 (16)	0.0303 (3)	
C7	0.6859 (2)	0.04029 (7)	0.78590 (16)	0.0303 (3)	
C8	0.01106 (19)	0.35845 (7)	0.60141 (15)	0.0281 (3)	
C9	−0.1341 (2)	0.33448 (7)	0.67764 (16)	0.0326 (3)	
H9	−0.2289	0.3622	0.7052	0.039*	
C10	−0.1397 (2)	0.26951 (8)	0.71351 (18)	0.0384 (4)	
C11	0.0036 (3)	0.23006 (8)	0.6699 (2)	0.0471 (4)	
H11	0.0023	0.1862	0.6914	0.057*	
C12	0.1487 (3)	0.25462 (8)	0.5951 (2)	0.0484 (5)	
H12	0.2440	0.2268	0.5686	0.058*	
C13	0.1568 (2)	0.31966 (8)	0.55812 (18)	0.0367 (4)	
C14	0.3146 (3)	0.34526 (9)	0.4753 (2)	0.0562 (5)	
H14A	0.2589	0.3651	0.3874	0.084*	
H14B	0.3971	0.3104	0.4524	0.084*	
H14C	0.3886	0.3767	0.5329	0.084*	
C15	−0.2965 (3)	0.24282 (9)	0.7963 (2)	0.0571 (5)	
H15A	−0.2783	0.1971	0.8094	0.086*	
H15B	−0.4198	0.2507	0.7433	0.086*	
H15C	−0.2917	0.2635	0.8890	0.086*	
N1	0.00494 (17)	0.42673 (5)	0.56098 (14)	0.0306 (3)	
H1A	−0.0346	0.4304	0.4673	0.046*	
H1B	0.1223	0.4438	0.5785	0.046*	
H1C	−0.0766	0.4475	0.6126	0.046*	
O1	0.84427 (15)	0.06900 (6)	0.78665 (13)	0.0441 (3)	
O2	0.65428 (15)	−0.00223 (6)	0.87917 (13)	0.0431 (3)	
O3	0.29811 (16)	−0.00571 (6)	0.79399 (13)	0.0469 (3)	
H3A	0.402 (2)	−0.0124 (11)	0.844 (2)	0.070*	

### Atomic displacement parameters (Å^2^)

**Table:**

	*U*^11^	*U*^22^	*U*^33^	*U*^12^	*U*^13^	*U*^23^
C1	0.0274 (7)	0.0264 (7)	0.0274 (7)	0.0019 (5)	0.0017 (6)	−0.0008 (5)
C2	0.0388 (8)	0.0367 (8)	0.0365 (8)	−0.0016 (7)	0.0067 (7)	0.0049 (7)
C3	0.0619 (12)	0.0442 (10)	0.0355 (9)	0.0061 (8)	0.0017 (8)	0.0117 (7)
C4	0.0468 (10)	0.0568 (11)	0.0337 (9)	0.0155 (8)	−0.0099 (7)	0.0011 (8)
C5	0.0291 (8)	0.0546 (10)	0.0365 (9)	0.0022 (7)	−0.0037 (7)	−0.0051 (7)
C6	0.0287 (7)	0.0336 (7)	0.0284 (7)	−0.0007 (6)	0.0021 (6)	0.0000 (6)
C7	0.0257 (7)	0.0345 (8)	0.0308 (7)	0.0014 (6)	0.0036 (6)	0.0004 (6)
C8	0.0281 (7)	0.0252 (7)	0.0296 (7)	0.0024 (5)	−0.0032 (6)	−0.0023 (6)
C9	0.0293 (7)	0.0318 (7)	0.0365 (8)	0.0021 (6)	0.0017 (6)	−0.0035 (6)
C10	0.0423 (9)	0.0350 (8)	0.0371 (8)	−0.0039 (7)	0.0005 (7)	0.0021 (7)
C11	0.0547 (11)	0.0282 (8)	0.0579 (11)	0.0044 (7)	0.0025 (9)	0.0050 (8)
C12	0.0450 (10)	0.0346 (9)	0.0659 (12)	0.0127 (7)	0.0066 (9)	−0.0058 (8)
C13	0.0338 (8)	0.0335 (8)	0.0431 (9)	0.0033 (6)	0.0050 (7)	−0.0046 (7)
C14	0.0482 (11)	0.0482 (10)	0.0763 (14)	0.0038 (8)	0.0272 (10)	−0.0080 (10)
C15	0.0622 (12)	0.0484 (11)	0.0629 (12)	−0.0095 (9)	0.0165 (10)	0.0084 (9)
N1	0.0274 (6)	0.0268 (6)	0.0370 (7)	0.0000 (5)	0.0003 (5)	−0.0015 (5)
O1	0.0278 (6)	0.0586 (7)	0.0452 (7)	−0.0091 (5)	−0.0002 (5)	0.0057 (6)
O2	0.0305 (6)	0.0545 (7)	0.0429 (6)	−0.0005 (5)	−0.0028 (5)	0.0203 (5)
O3	0.0300 (6)	0.0659 (8)	0.0436 (7)	−0.0128 (5)	−0.0013 (5)	0.0194 (6)

### Geometric parameters (Å, º)

**Table:**

C1—C2	1.388 (2)	C9—H9	0.9300
C1—C6	1.399 (2)	C10—C11	1.381 (2)
C1—C7	1.4886 (19)	C10—C15	1.502 (3)
C2—C3	1.378 (2)	C11—C12	1.379 (3)
C2—H2	0.9300	C11—H11	0.9300
C3—C4	1.379 (3)	C12—C13	1.392 (2)
C3—H3	0.9300	C12—H12	0.9300
C4—C5	1.371 (2)	C13—C14	1.500 (2)
C4—H4	0.9300	C14—H14A	0.9600
C5—C6	1.386 (2)	C14—H14B	0.9600
C5—H5	0.9300	C14—H14C	0.9600
C6—O3	1.3556 (18)	C15—H15A	0.9600
C7—O1	1.2524 (17)	C15—H15B	0.9600
C7—O2	1.2696 (18)	C15—H15C	0.9600
C8—C9	1.383 (2)	N1—H1A	0.8900
C8—C13	1.385 (2)	N1—H1B	0.8900
C8—N1	1.4615 (18)	N1—H1C	0.8900
C9—C10	1.387 (2)	O3—H3A	0.829 (10)
			
C2—C1—C6	118.59 (13)	C9—C10—C15	121.27 (15)
C2—C1—C7	120.85 (13)	C12—C11—C10	121.15 (15)
C6—C1—C7	120.55 (13)	C12—C11—H11	119.4
C3—C2—C1	121.10 (15)	C10—C11—H11	119.4
C3—C2—H2	119.4	C11—C12—C13	122.07 (15)
C1—C2—H2	119.4	C11—C12—H12	119.0
C2—C3—C4	119.33 (16)	C13—C12—H12	119.0
C2—C3—H3	120.3	C8—C13—C12	116.06 (15)
C4—C3—H3	120.3	C8—C13—C14	122.70 (14)
C5—C4—C3	120.96 (15)	C12—C13—C14	121.23 (15)
C5—C4—H4	119.5	C13—C14—H14A	109.5
C3—C4—H4	119.5	C13—C14—H14B	109.5
C4—C5—C6	119.82 (15)	H14A—C14—H14B	109.5
C4—C5—H5	120.1	C13—C14—H14C	109.5
C6—C5—H5	120.1	H14A—C14—H14C	109.5
O3—C6—C5	118.17 (13)	H14B—C14—H14C	109.5
O3—C6—C1	121.70 (12)	C10—C15—H15A	109.5
C5—C6—C1	120.12 (14)	C10—C15—H15B	109.5
O1—C7—O2	122.51 (13)	H15A—C15—H15B	109.5
O1—C7—C1	119.68 (13)	C10—C15—H15C	109.5
O2—C7—C1	117.81 (12)	H15A—C15—H15C	109.5
C9—C8—C13	122.38 (14)	H15B—C15—H15C	109.5
C9—C8—N1	118.31 (12)	C8—N1—H1A	109.5
C13—C8—N1	119.27 (13)	C8—N1—H1B	109.5
C8—C9—C10	120.71 (14)	H1A—N1—H1B	109.5
C8—C9—H9	119.6	C8—N1—H1C	109.5
C10—C9—H9	119.6	H1A—N1—H1C	109.5
C11—C10—C9	117.62 (15)	H1B—N1—H1C	109.5
C11—C10—C15	121.10 (15)	C6—O3—H3A	106.5 (16)
			
C6—C1—C2—C3	0.3 (2)	C6—C1—C7—O2	10.7 (2)
C7—C1—C2—C3	−178.85 (15)	C13—C8—C9—C10	−0.4 (2)
C1—C2—C3—C4	1.8 (3)	N1—C8—C9—C10	177.30 (13)
C2—C3—C4—C5	−1.7 (3)	C8—C9—C10—C11	−0.2 (2)
C3—C4—C5—C6	−0.6 (3)	C8—C9—C10—C15	−179.99 (15)
C4—C5—C6—O3	−178.60 (15)	C9—C10—C11—C12	0.9 (3)
C4—C5—C6—C1	2.7 (2)	C15—C10—C11—C12	−179.39 (17)
C2—C1—C6—O3	178.81 (14)	C10—C11—C12—C13	−0.9 (3)
C7—C1—C6—O3	−2.0 (2)	C9—C8—C13—C12	0.4 (2)
C2—C1—C6—C5	−2.6 (2)	N1—C8—C13—C12	−177.30 (14)
C7—C1—C6—C5	176.58 (14)	C9—C8—C13—C14	179.91 (15)
C2—C1—C7—O1	10.0 (2)	N1—C8—C13—C14	2.3 (2)
C6—C1—C7—O1	−169.13 (14)	C11—C12—C13—C8	0.3 (3)
C2—C1—C7—O2	−170.18 (14)	C11—C12—C13—C14	−179.29 (17)

### Hydrogen-bond geometry (Å, º)

**Table:**

*D*—H···*A*	*D*—H	H···*A*	*D*···*A*	*D*—H···*A*
O3—H3*A*···O2	0.83 (1)	1.77 (1)	2.5282 (15)	151 (2)
N1—H1*A*···O1^i^	0.89	1.80	2.6809 (17)	169
N1—H1*B*···O2^ii^	0.89	1.92	2.7998 (16)	168
N1—H1*C*···O3^iii^	0.89	2.08	2.9654 (17)	171
C5—H5···O1^iv^	0.93	2.58	3.237 (2)	128

Symmetry codes: (i) *x*−1, −*y*+1/2, *z*−1/2; (ii) −*x*+1, *y*+1/2, −*z*+3/2; (iii) −*x*, *y*+1/2, −*z*+3/2; (iv) *x*−1, *y*, *z*.
