# Supplementary figures and images for: Crystal structure of 2,5-di­methyl­anilinium salicylate
Source: Acta Crystallogr E Crystallogr Commun. 2015 Aug 6;71(Pt 9):o643–4. doi: 10.1107/S2056989015014401 (PMC4555438; doi:10.1107/S2056989015014401)

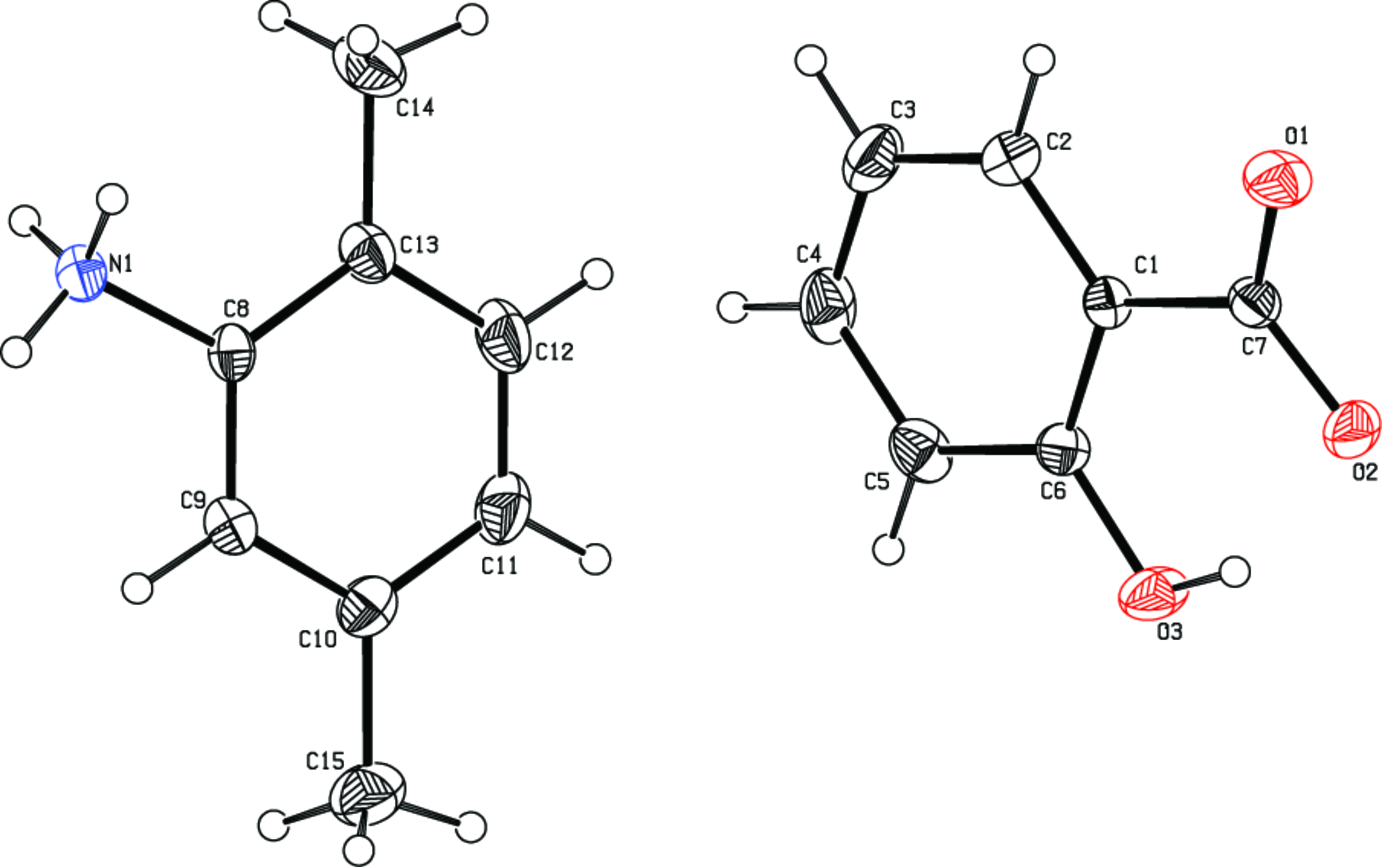

Supplement: Supplementary file 4 [file e-71-0o643-fig1.tif]

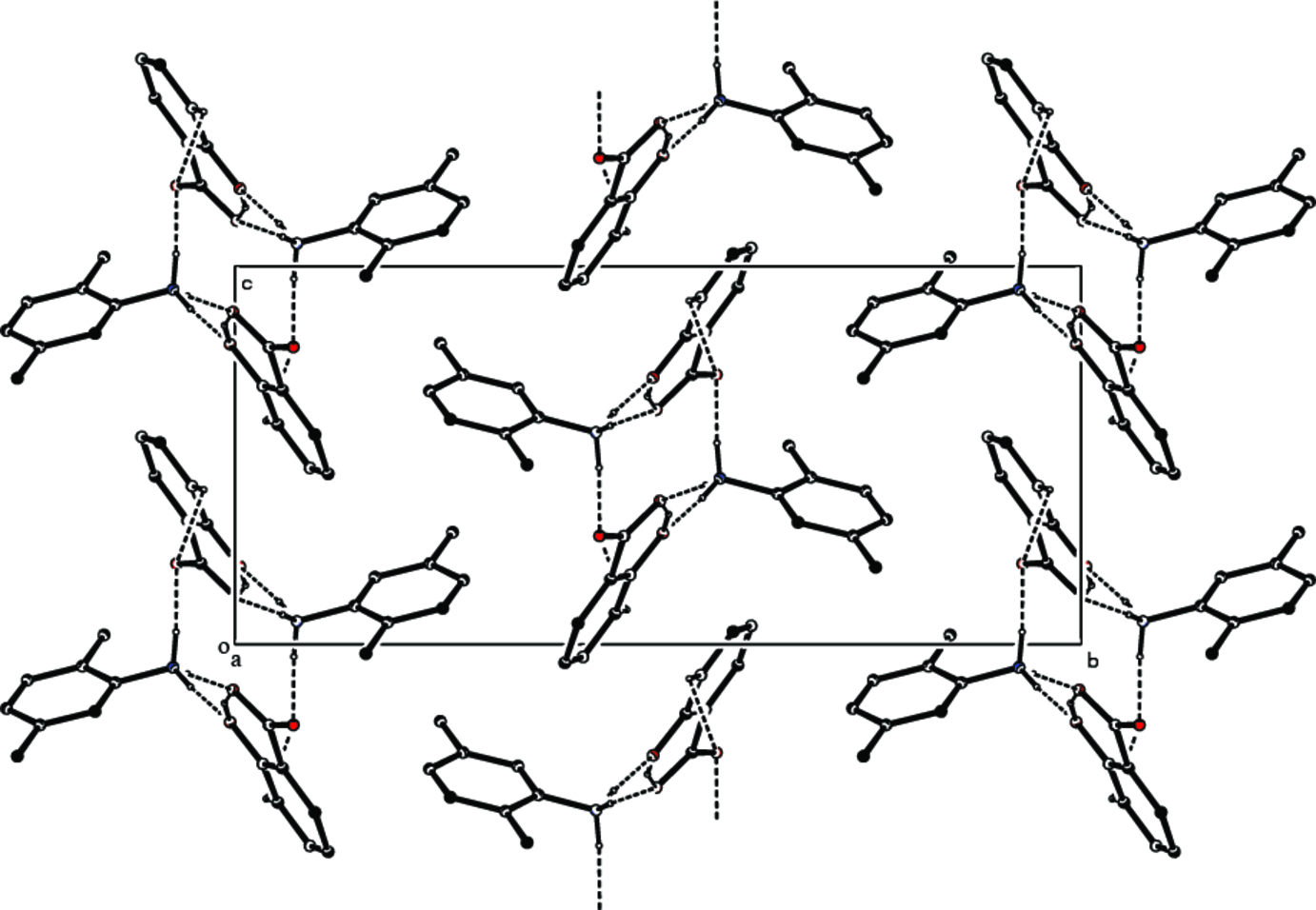

Supplement: Supplementary file 5 [file e-71-0o643-fig2.tif]
